# Coronal malalignment of lower legs depending on the locations of the exostoses in patients with multiple hereditary exostoses

**DOI:** 10.1186/s12891-019-2912-6

**Published:** 2019-11-25

**Authors:** Yeong Seub Ahn, Seong Hwan Woo, Sung Ju Kang, Sung Taek Jung

**Affiliations:** Department of Orthopedic Surgery, Chonnam National University Hospital, 42 Jebongro, Donggu, Gwangju, 501-757 Republic of Korea

**Keywords:** Ankle valgus, Multiple hereditary exostoses, Location of exostosis

## Abstract

**Backgrounds:**

Though malalignment of lower legs is a common pathologic phenomenon in multiple hereditary exostoses (MHE), relationship between locations of exostoses and malalignment of lower legs remains unclear. This study examined radiographs of MHE patients in an attempt to evaluate the tendency of coronal malalignment of lower legs with different location of exostoses on lower legs consisting of two parallel long bones.

**Methods:**

Between 2000 and 2017, we retrospectively reviewed the anteroposterior films of the teleo-roentgenographics of 63 patients with MHE. The patients were classified into four different groups depending on the locations of the exostosis, which occurred on both proximal and distal tibiofibular joints (A), proximal tibiofibular joint (B), distal tibiofibular joint (C), and not for the tibiofibular joint area (D). To evaluate the influence of the location of exostoses on coronal malalignment of lower legs, medial proximal tibia angle (MPTA), lateral distal tibia angle (LDTA), and fibular shortening were analyzed for each group.

**Results:**

Significant difference was observed in multiple comparative analyses for each of the four groups. On MPTA radiologic analysis, group A showed greatest value with significant difference compared with groups C and D (vs. (B): *p* = 0.215; vs. distal joints (C): *p* = 0.004; vs. (D): *p* = 0.001). Group B showed significant difference only with group D (vs. distal joints (C): *p* = 0.388; vs. (D): *p* = 0.002), but for group C and D showed no significant difference. For LDTA, only group A showed significant difference compared to other groups (*p* < 0.001). With regard to tibiofibular ratio for evaluation of fibular shortening, group A showed the lowest ratio (vs. (B): *p* = 0.004; vs. (C): *p* = 0.655; vs. (D): p < 0.001). Group C also presented the significant lower ratio than group D (*p* = 0.002).

**Conclusions:**

For evaluation of the coronal malalignment of lower legs in MHE patients, not only ankle around the distal tibiofibular joint but also proximal tibiofibular joint should be examined, in that, lower limb deformity occurred by two parallel long bone which has self-contained joint.

**Level of evidence:**

Level III, retrospective comparative study.

## Background

Multiple hereditary exostoses (MHE), with an estimated frequency of at least once per 50,000, is one of the most common bony dysplasia occurring in the metaphysis of bones developed by endochondral ossification [1, 2]. The most common symptom of MHE is presence of palpable mass or limitation of range of motion, and rarely malignant change [3]. In addition, most of the patients with MHE have a high tendency of developing bony deformities in the extremities. The growths may alter bony development and lead to angular deformity with or without subsequent length discrepancies. The relative shortening of the fibula and obliquity of the distal tibia epiphysis results in valgus deformities of the ankle [4–7]. Moreover, osteochondromas in distal tibiofibular articular area may impart a tethering effect to lateral growth in distal joint, resulting in valgus deformity in the ankle [8]. Several authors have reported ankle valgus deformities in distal tibiofibular articular area around the ankle joint in patients with osteochondroma [9–11]. However, in terms of the prevalence of MHE, most of the common lesions were reported around the knee joint [2]. Considering that the lower legs below the knee joint consist of two parallel long bones (such as forearms), which influence bony growth, proximal articular area should also be considered as an important factor for review of deformities of ankle valgus presented as tibia bowing. Until now, there exists no report about coronal malalignment in MHE involving both the lower legs.

We postulated that tendency of coronal malalignment in the lower leg might be influenced by the locations of exostosis including proximal tibiofibular articular area around the knee joint as well as distal tibiofibular articular area around the ankle joint in view of tethering factor between the two parallel bones.

Apparently, the aim of this study was to evaluate coronal malalignment along with a thorough evaluation of the lower legs, distal tibiofibular articular area around the ankle joint, and proximal tibiofibular articular area around the knee joint using anteroposterior teleoroentgenographic radiographics.

## Methods

This research was conducted through a retrospective review of patients diagnosed with MHE from Jan 1, 2001, to Jan 1, 2017. Inclusion criteria for this study were patients with MHE who had radiographs of anteroposterior teleo-roentgenographics, in particular, who had any radiograph before surgical intervention or had not undergone any surgical intervention. Moreover, retrospective of multi-group comparative study involving a tertiary referral center was approved. Exclusion criteria included inadequate radiographic findings. In addition, patients who visited initially with operative state at other departments were excluded.

**Fig. 1:**
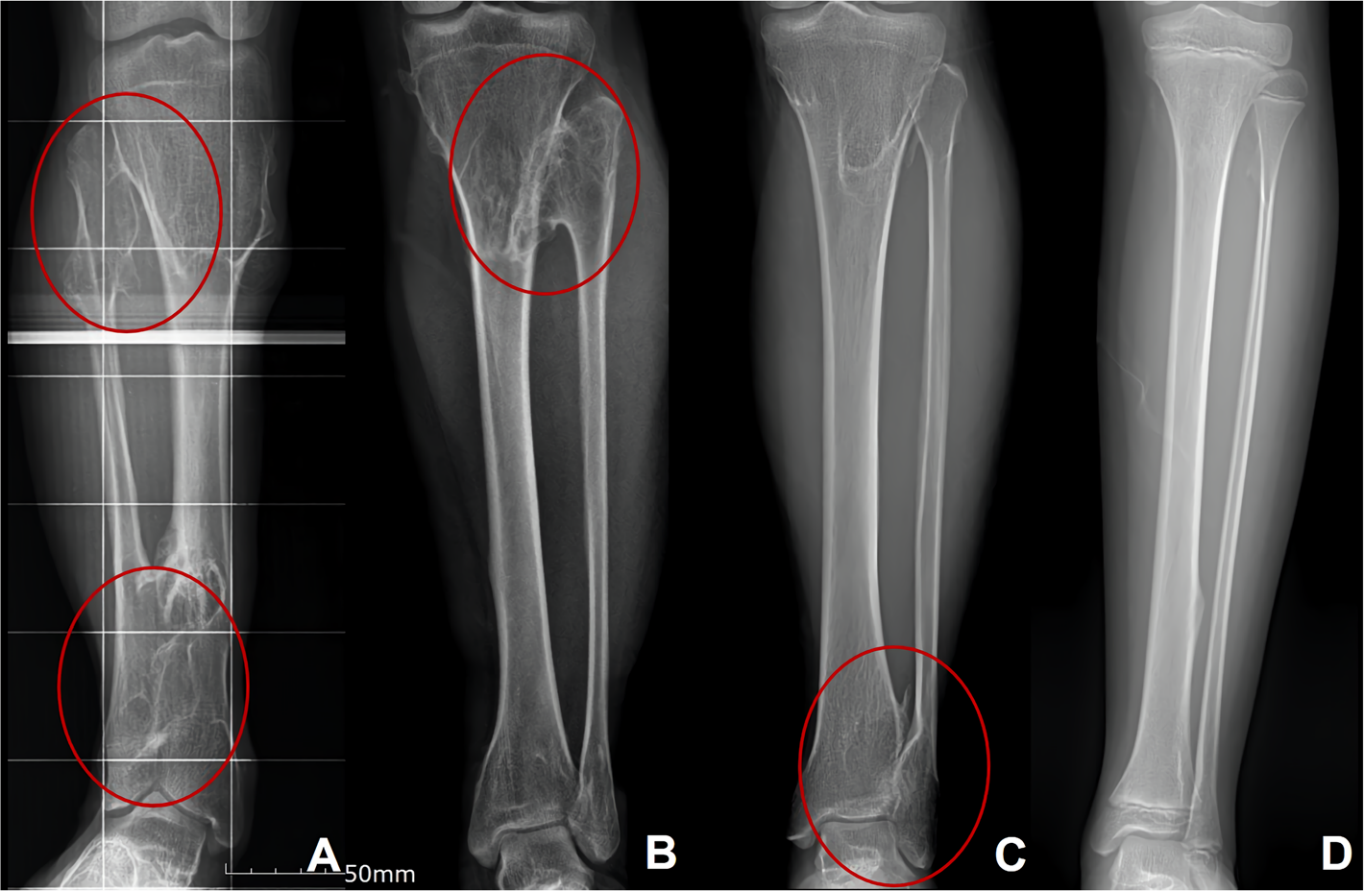
Method of classification of the lower leg of MHE patients depending on the location of the exostosis. Figure A shows a patient in group A who have lesion of exostoses both the proximal and distal tibiofibular joints of the lower leg. Figure **b** shows the patient classified as group B that only have the exostoses involving the proximal tibiofibular joint of the lower leg. Figure **c** shows a radiograph of a group C patient with only the distal tibiofibular joint invasion. Figure **d** shows a group D that have lesion without both proximal and distal joint involvement

All the patients within inclusion criteria were classified into four different groups depending on the locations of the exostosis. The patients with exostoses who had both proximal and distal tibiofibular articular joints area were classed as group A, proximal tibiofibular joint area as group B, distal tibiofibular joint area as group C, and absence of tibiofibular joint area as group D (Fig. 1).

### Radiographic analysis

**Fig. 2:**
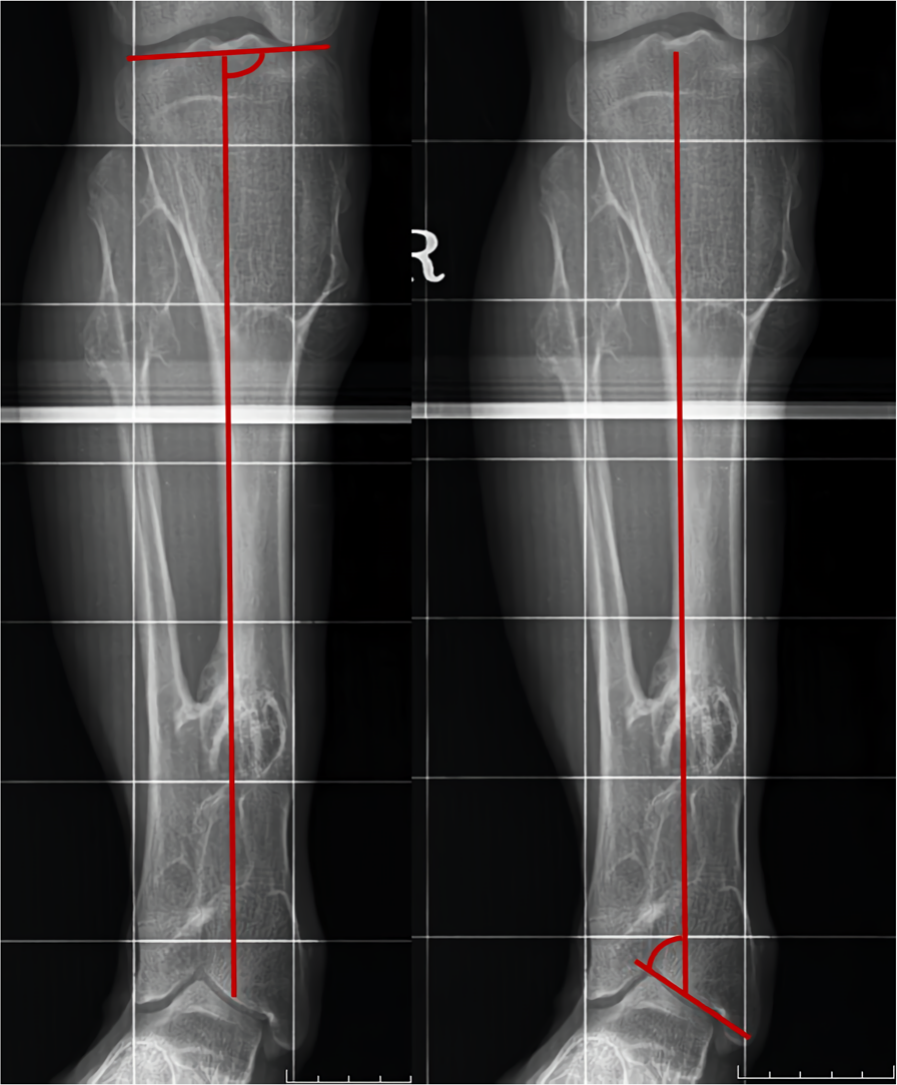
Measurement of radiographic angle of MPTA and LDTA. The MPTA is determined by measuring the angle created by a line of the central axis of the tibia and a second line drawn across the proximal tibial epiphyseal surface or joint line. In a similar way, LDTA is measured by the angle created by a line of central axis of the tibia and a second line drawn across the distal tibial epiphyseal surface or talar plafond

For non-operative patients, radiographic analysis was conducted using the last follow-up radiographs. On the other hand, for patients who underwent any surgical intervention were evaluated using last follow-up preoperative radiographs. In addition, the radiographic analysis was performed for all the four groups. To measure the angulation of lower leg, in particular, around knee and ankle joint, medial proximal tibial angle (MPTA) and lateral distal tibial angle (LDTA) were evaluated (Fig. 2).

**Fig. 3:**
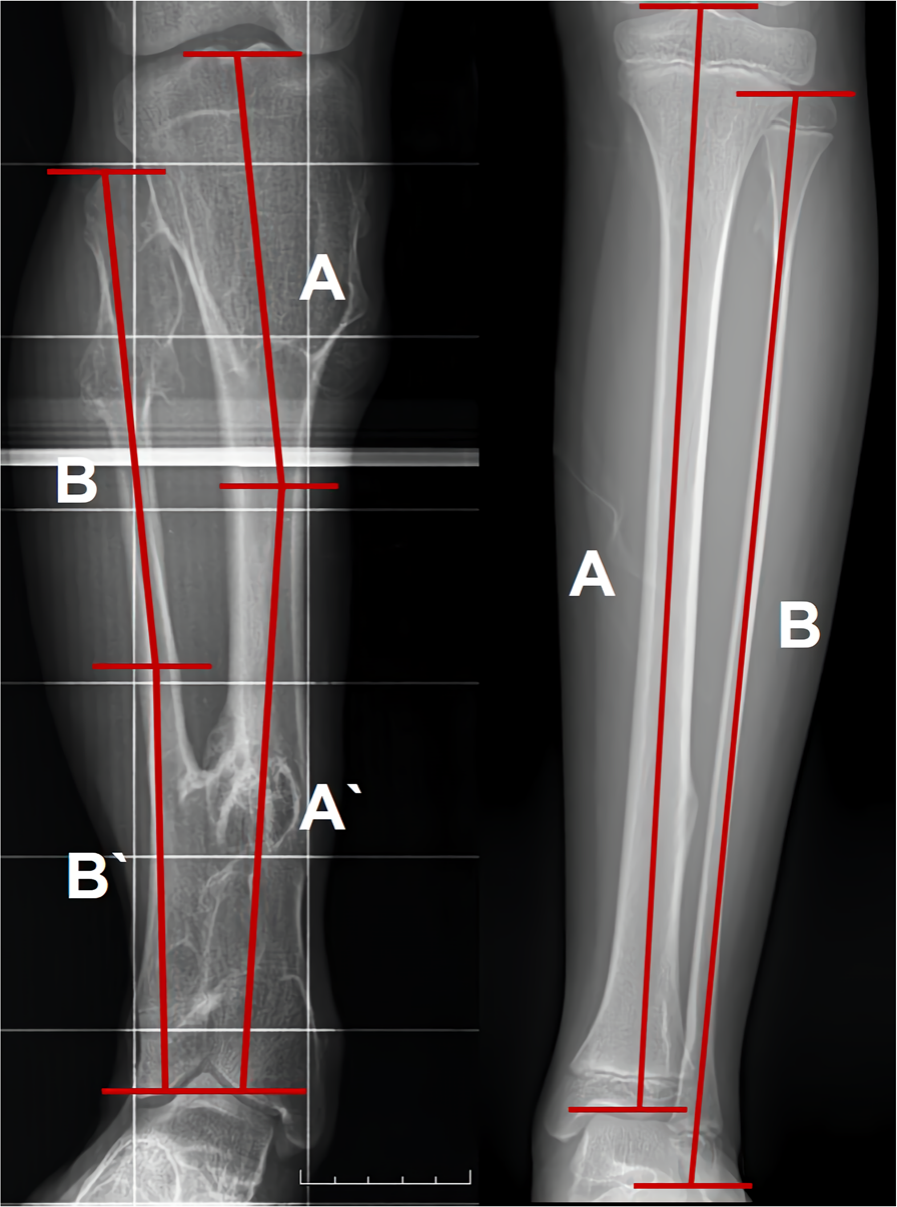
Method of the measurement of fibular shortening based on relative fibular length to the tibia. Relative fibular length to the tibia is determined by comparing longitudinal line from the top to base. In severe case of angular deformity, two longitudinal lines from top and base were measured and summed the distance from the intersection point

To measure the fibular shortening, relative fibular/tibia length was evaluated. Relative fibular length to the tibia is determined by comparing each of longitudinal line from the top to base. If angular deformity was severe, two longitudinal lines from top and base were measured and summed the distance from the intersection point (Fig. 3).

### Statistical analysis

Statistical analysis was carried out using the SPSS software. The Kruskal-Wallis test and Tukey test were applied to compare the four different groups based on the location of exostosis. Statistical significance was ascribed to *P* < 0.05.

## Results

Among 70 patients diagnosed with MHE who visited our department (Chonnam National University Hospital Orthopedic department) from Jan 2001 to Jan 1, 2017, 63 patients with 126 limbs were sorted out. Five patients who had no radiographs of pre-operative state and 2 patients with inadequate quality were excluded. Total 63 patients with 126 limbs were classified into four groups for evaluating the relationship between tendencies of lower limb valgus deformity and location of exostosis. The mean age at the time of last radiographic record of the four groups is summarized in Table 1; no significant differences in these values were noted.

**Table 1:** Mean age at last radiographic record of each group

	Number of lower legs	Mean age, y
Group A	51	14.5 (range, 4.4 to 36.0)
Group B	29	10.9 (range, 4.4 to 20.8)
Group C	20	12.6 (range, 4.7 to 32.1)
Group D	26	11.4 (range, 4.1 to 32.1)

Significant results were obtained from the Kruskal-Wallis test on evaluation of MPTA, LDTA, and fibular shortenings among the four groups. Moreover, the Tukey test was performed to find significant differences between comparisons of each group.

In terms of MPTA radiographic analysis implying valgus deformity around the knee joint, group A showed the greatest value followed by group B, C, and D (Table 2). And group A exhibited significant difference than the other two groups (vs. (B): *p* = 0.215; vs. distal joints (C): *p* = 0.004; vs. (D): *p* = 0.001). Though proximal group B showed more valgus implying values than distal group C, there was no significant difference between the two groups (vs. distal joints (C): *p* = 0.388). However group B showed significant difference than group D (vs. (D): *p* = 0.002), whereas, group C showed no significant changes in values compared with group D. Consequently, group A showed most valgus implying values on knee joint, and proximal group B appeared to be more influencing on proximal tibiofibular joint than distal group C compiling MPTA radiologic values (Table 3).

**Table 2:** Radiographic assessment of four groups classified based on the location of bony exostosis site on the lower leg

	Group A (Both)		Group B (Proximal)		Group C (Distal)		Group D (Others)	
	Mean	SD	Mean	SD	Mean	SD	Mean	SD
MPTA	93.43°	3.46°	92.26°	2.01°	91.08°	1.46°	89.67°	1.45°
LDTA	78.96°	6.54°	86.10°	2.76°	85.22°	3.74°	88.78°	2.14°

Considering the value of both MPTA and LDTA as a reflection of genu valga and ankle valgus deformity for each, group A seemed to have a tendency of valgus deformity at most in knee and ankle joints

On analysis of MPTA radiographic analysis implying valgus deformity around knee joint, group A showed the greatest value followed by group B, C, and D. With LDTA radiographic analysis suggesting valgus deformity on ankle joint, group A showed the lowest value in the degree of ankle valgus deformity. The next following groups were C, B, and D in decreasing order

**Table 3:** *P*-value of intergroup difference in MPTA

Radiographic Index	Comparison between the groups		*P*-value
MPTA	A (Both)	B	0.215
		C	0.004
		D	0.001
	B (Proximal)	A	0.215
		C	0.388
		D	0.002
	C (Distal)	A	0.004
		B	0.388
		D	0.263
	D (Neither)	A	0.001
		B	0.002
		C	0.263

Group A seemed to have significant difference than the two groups (vs. (B): p = 0.215; vs. distal joints (C): p = 0.004; vs. (D): p = 0.001). Though group B showed more valgus implying values than group C, there was no significant difference between the two groups (vs. (C): p = 0.388). But group B showed a significant difference than the group D (vs. (D): p = 0.002), and group C showed no changes in values compared with group D

On LDTA radiographic analysis, which suggested valgus deformity of ankle joint, group A showed the lowest value in the degree of ankle valgus deformity. Following were the groups C, B, and D in a decreasing trend (Table 2). Group A was the only group which showed significant result with the *p*-value lower than 0.001. However, *p*-value of other three groups except for group A showed no significant difference (Table 4).

**Table 4:** *P*-value of intergroup differences in LDTA

Radiographic Index	Comparison between the groups		*P*-value
LDTA	A (Both)	B	0.000
		C	0.000
		D	0.000
	B (Proximal)	A	0.000
		C	0.939
		D	0.139
	C (Distal)	A	0.000
		B	0.939
		D	0.060
	D (Neither)	A	0.000
		B	0.139
		C	0.060

Group A was the only group, which showed a significant result, the *p*-value lower than 0.001. However, *p*-value of other three groups except for group A showed no significant difference. Although significant difference was not observed except for group A, group C was thought to be affected more than group B with regard to LDTA value

Fibular shortening was evaluated for each group based on the relative length of tibia and fibula axis. On tibiofibular ratio analysis, group A showed the lowest ratio followed by group C, B, and D (Table 5). Group A showed significant difference compared to other groups except for group C (vs. (B): *p* = 0.004; vs. (C): *p* = 0.655; vs. (D): *p* < 0.001). Moreover, group C presented the lower ratio than other groups except for group A and showed significantly lower ratio compared to group D (*p* = 0.002) (Table 6).

**Table 5:** Relative fibula length

Group	Relative fibular length	
	Mean	SD
A	0.9572	0.0265
B	0.9747	0.0176
C	0.9639	0.0215
D	0.9874	0.0126
Total	0.9686	0.0243

On tibiofibular ratio analysis, group A showed the lowest ratio followed by group C, B, and D

**Table 6:** *P*-value of intergroup difference on fibular shortening

Radiographic Index	Comparison between the groups		*P*-value
Fibular shortening (Fibula/Tibia length)	A (Both)	B	0.004
		C	0.655
		D	0.000
	B (Proximal)	A	0.004
		C	0.307
		D	0.137
	C (Distal)	A	0.655
		B	0.307
		D	0.002
	D (Neither)	A	0.000
		B	0.137
		C	0.002

Group A showed significant difference compared to other groups except for group C (vs. (B): p = 0.004; vs. (C): p = 0.655; vs. (D): p < 0.001). Moreover, group C presented the lower ratio than other groups except for group A and showed significant lower ratio compared to group D (p = 0.002)

## Discussion

MHE is a congenital autosomal dominant disorder as proven by geno-phenotype research of EXT gene family [12–15], which is involved in the regulation of cartilage synthesis most often originating at the metaphysis of long bones. Among the commonly stated symptoms of MHE, cartilage capped bony growth resulting in angular deformity is the typical characterization. Numerous studies have reported the manner in which the lesions around the distal tibiofibular articulation area influence ankle deformities and subsequently resulting in hypoplasia of the lateral aspect of the distal tibia epiphysis and valgus deformation in patients of MHE [6, 7, 16].

Among the commonly noted studies on the occurrence of MHE on lower legs, Schemale et al. [2] reported occurrence of MHE lesions in the proximal tibia in approximately 70% of patients, and knee joint as the most commonly involved region. In terms of bony growth, the growth around the knee is the major phenomenon and the knee accounts for about two-thirds of the growth in the lower limbs [17, 18]. Considering the research about occurrence rate of osteochondroma or exostoses in the lower extremities [2] and the growth potential of lower extremity around the knee joint account for which is the largest in lower extremities, the only distal tibiofibular articular lesion has a limitation to provide an explanation of ankle valgus deformity. In view of anatomical morphology of lower legs constructed in two parallel long bones affecting bony growth to each other, both the lesions around the distal and proximal tibiofibular articular area should be included to evaluate the coronal malalignment of lower leg in patients with MHE.

This study focuses on the severity of the malalignment of the lower limb depending on the location of the exostoses. Despite the location of tumors at various lesions in the lower limb, the overall tendency of the malalignment in MHE was at the site of valgus deformity.

In view of the tendency of valgus deformity, group B with proximal tibiofibular joint showed more valgus tendency on MPTA and group C with distal tibiofibular joint showed more valgus tendency on LDTA. Moreover, group A with both proximal and distal tibiofibular joint showed the most valgus tendency around the ankle joint as well as the knee joint.

Fibular shortening was also most prominent in group A followed by group C, B, and D. By combining the results, it becomes apparent that Group A showed significant difference compared to other groups except for group C and group C showed significant difference than group D. Both proximal and distal joint combined lesion had utmost impact on fibular shortening followed by distal joint involved lesion.

The obtained results could be interpreted comprehensively by stating that MHE occurring at the proximal and distal tibiofibular joint area has more influence on malalignment of each joint. However, considering the result of group A that both proximal and distal tibiofibular involved MHE have utmost coronal malalignment of lower leg.

However, further evaluation of the correlation between the osteochondroma development and angular deformity needs to be investigated as osteochondroma developing diaphyseal or metaphyseal area can also affect angular deformities. Moreover, delicated impact of exostoses affecting angular deformity was not evaluated. Hence, this study has some limitations in that it focuses only on malalignment by exsostoses location around the joint.

## Conclusions

This study reveals that the tendency of ankle valgus deformities might be influenced by the locations of exostosis on lower legs including the proximal tibiofibular articular area around the knee joint and distal tibiofibular articular area around the ankle joint. In particular, when both the proximal and distal tibiofibular joints were involved, malalignment seemed to be more severe. Therefore, for evaluation of ankle valgus deformity in MHE patients, not only ankle around the distal tibiofibular joint but also proximal tibiofibular joint should be examined, in that, lower limb deformity occurred by two parallel long bone which has self-contained joint. In addition, the patients with involvement of both proximal and distal tibiofibular joints should be extensively followed up or managed.

## Data Availability

The datasets used and/or analyzed during the current study are available from the corresponding author on reasonable request.

## Abbreviations

LDTA: Lateral distal tibia angle
MHE: Multiple hereditary exostoses
MPTA: Medial proximal tibia angle

## References

[1] Noonan KJ, Feinberg JR, Levenda A (2002). Natural history of multiple hereditary osteochondromatosis of the lower extremity and ankle. J Pediatr Orthop.

[2] Schmale GA, Conrad EU, Raskind WH (1994). The natural history of hereditary multiple exostoses. J Bone Joint Surg Am.

[3] Porter DE, Lonie L, Fraser M (2004). Severity of disease and risk of malignant change in hereditary multiple exostoses. A genotype-phenotype study. J Bone Joint Surg Br..

[4] Dias LS (1985). Valgus deformity of the ankle joint : pathogenesis of fibular shortening. J Pediatr Orthop.

[5] Jahss MH, Olives R (1980). The foot and ankle in multiple hereditary exostoses. Foot Ankle.

[6] Snearly WN, Peterson HA (1989). Management of ankle deformities in multiple hereditary osteochondroma. J Pediatr Orthop.

[7] Solomon L (1961). Bone growth in diaphysealaclasis. J Bone Joint Surg Br..

[8] Taniguchi K (1995). A pracitical classification system for multiple cartilaginousexostosis in children. J Pediatr Orthop.

[9] Martin R, Alexander SS, Carsten S, Sandra B, Karsten R, Ralf S (2015). Rebound ankle valgus deformity in patients with hereditary multiple exostosis. J Pediar Orthop.

[10] Matthew D, Judith L, Elroy S, Allison S (2013). Correction and recurrence of ankle valgus in skeletally immature patients with multiple hereditary exostoses. Foot Ankle Int.

[11] Takikawa K, Haga N, Tanaka H, Okada K (2008). Characteristic factors of ankle valgus with multiple cartilaginous exostoses. J Pediatr Orthop.

[12] Philippe C, Porter DE, Emerton ME (1997). Mutation screening of EXT 1 and EXT 2 genes in patients with hereditary multiple exostoses. Am J Hum Genet.

[13] Raskind WH, Conrad EU, Matsushita M (1998). Evaluation of locus heterogeneity and EXT 1 mutations in 34 families with hereditary multiple exostoses. Hum Mutant.

[14] Wells DE, Hill A, Lin X (1997). Identification of novel mutations in the human EXT1 tumor suppressor gene. Hum Genet.

[15] Wuyts W, Van Hul W, De Boulle K (1998). Mutations in the EXT1 and EXT2 genes in hereditary multiple exostoses. Am J Hum Genet.

[16] Malhotra D, Pau R, Owen R (1984). Valgus deformity of the ankle in children with spina bifida aperta. J Bone Joint Surg Br.

[17] Anderson M, Green WT, Messener MB (1963). Growth and predictions of growth in the lower extremities. J Bone Joint Surg Am.

[18] Dimeglio A (2001). Growth in pediatric orthopaedics. J Pediatric Orthop.

